# Hypnosis to reduce fear of falling in hospitalized older adults: a feasibility randomized controlled trial

**DOI:** 10.1186/s40814-023-01366-3

**Published:** 2023-08-09

**Authors:** Clémence Cuvelier, Mélany Hars, Maria Pia Zamorani-Bianchi, François R. Herrmann, Catherine Ducharne Wieczorkiewicz, Dina Zekry, Gabriel Gold, Andrea Trombetti

**Affiliations:** 1 Division of Geriatrics, Department of Rehabilitation and Geriatrics, Geneva University Hospitals and Faculty of Medicine, Thônex, Switzerland; 2 Division of Bone Diseases, Department of Medicine, Geneva University Hospitals and Faculty of Medicine, Geneva, Switzerland; 3 Division of Internal Medicine for the Aged, Department of Rehabilitation and Geriatrics, Geneva University Hospitals and Faculty of Medicine, Geneva, Switzerland

**Keywords:** Fear of falling, Hypnosis, Rehabilitation, Feasibility study, Older adults

## Abstract

**Background:**

Fear of falling is associated with numerous negative health outcomes in older adults and can limit the rehabilitation process. Hypnosis is now recognized as an effective treatment for a variety of conditions, especially anxiety and pain, which can be integrated safely with conventional medicine. The objective of this study was to assess the feasibility and acceptability of a hypnosis intervention in hospitalized older adults to reduce fear of falling.

**Methods:**

In this feasibility randomized controlled trial, 32 older patients, hospitalized in geriatric rehabilitation wards, were randomly allocated (1:1 ratio) to either an intervention group (hypnosis, 2 sessions, one per week, plus usual rehabilitation program) or a control group (usual rehabilitation program only). Clinical assessors and statistician were blinded to group allocation. Primary outcomes were recruitment rate, retention rate, and adherence to the intervention. Exploratory outcomes, analyzed according to the intention-to-treat principle, included impact of hypnosis on fear of falling (assessed by a new scale perform-FES), functional status, in-hospital falls, and length of hospital stay.

**Results:**

Recruitment rate was 1.3 patients per week. The recruitment of the population sample was achieved in 5.5 months. The retention rate did not differ significantly between groups and a good adherence to the hypnosis intervention was achieved (77% of patients received the full intervention). No adverse event related to the hypnosis intervention was observed. Regarding exploratory clinical outcomes, no differences were found between groups on any outcome.

**Conclusion:**

Hypnosis is feasible and well accepted in a geriatric hospitalized population undergoing rehabilitation. Further pilot work should be conducted, with an increased number of hypnosis sessions, before conducting a full-scale trial to conclude whether, or not, hypnosis is effective to reduce fear of falling.

**Trial registration:**

NCT04726774.

## Key messages regarding feasibility

• What uncertainties existed regarding the feasibility?
  Whether hypnosis could be an effective intervention strategy to reduce fear of falling in older adults remains to be determined. We conducted a feasibility randomized controlled trial to address if a hypnosis intervention would be feasible and acceptable, and explore effectiveness, to inform a potential future large-scale study. Because this study focused on hospitalized very old patients, including patients with cognitive impairments, uncertainties existed especially regarding recruitment, feasibility, and adherence.
• What are the key feasibility findings?
  Hypnosis was feasible to deliver and well accepted by old patients hospitalized in a geriatric hospital. No adverse event related to the hypnosis intervention was observed.
• What are the implications of the feasibility findings for the design of the main study?
  The findings of this study provide important information about recruitment, adherence, and impact of a hypnosis intervention for a larger future randomized controlled trial for fear of falling reduction in hospitalized older adults. But further pilot work should be conducted, with an increased number of hypnosis sessions to demonstrate a clinical effect.

## Background

Falls are highly prevalent in older adults. They can result in serious injuries and death [1]. They can also have psychological consequences, such as fear or falling [2], which may lead to restriction of activities resulting in further worsening in functional performances, reduction of quality of life and loss of independence [3]. The relationship between falls and fear of falling is close, and fear of falling and falls share risk factors [4]. Fear of falling further increases the risk of falling [4]. Fear of falling can be the consequence of a rational appraisal of reduced functional abilities, or can be a construct reflecting the original phobic condition and may be irrational [5]. Its prevalence is estimated between 25 and 85% in older people according to settings and type of measure [6, 7], and fear of falling can also be present among those who never experienced falls [8]. Main fear of falling related risk factors include older age, female sex, functional impairment, or medications [9, 10]. In an inpatient geriatric rehabilitation population, fear of falling has been associated with worse functional recovery, more in-hospital falls, and increased length of stay [10–12]. Moreover, the high level of perceived fall risk is likely to be associated with future falls, independent of physiological risk [13]*,* and may influence physical capabilities, especially gait performances [8]. Considering this, strategies aimed at reducing fear of falling should be implemented in rehabilitation programs. In a meta-analysis [14] exercise interventions were associated with a small or moderate reduction in fear of falling in the short and long-term follow-up. Cognitive behavioural therapy has also been shown to reduce fear of falling in older adults [15], especially in combination with exercise [14, 16–18]. Studies suggested that multifactorial assessment and combinations of interventions reduce the risk of falls in older population [19, 20].

Fear of falling can be evaluated using different tools, mainly validated in the community and not in hospitalized population. Whereas some tools measure the ability to avoid a fall (i.e., Geriatric Fear of Falling Measure, GFFM) [21], or the confidence in maintaining balance (i.e., Activities-specific Balance Confidence scale, ABC) [22], other assess the concern about falling during activities (i.e., Fall Efficacy Scale-International, FES-I) [23]. This variety and heterogeneity of constructs can explain the difficulty to measure the impact of interventions on fear of falling [14, 24]. Moreover, these tools are based on answers to a questionnaire and may not reflect a person’s feelings during the actual functional performance. The answers may be affected by some degree of cognitive deficit and not really adapted to evaluate short term evolution, in which the subject is not reexposed to the situations depicted in the questionnaire. In that context and for the purpose of the current study, we developed the Perform-FES scale specifically designed to measure the degree of concern about falling in a hospital setting [25]. This scale demonstrated excellent psychometric properties.

Hypnosis is defined as “a state of consciousness involving focused attention and reduced peripheral awareness characterized by an enhanced capacity for response to suggestion” [26]. During a hypnotic trance, physiological, cognitive, and affective processes can be modified. Several hypnosis techniques, such as medical hypnosis, hypnotic communication, and hypnotherapy, are recognized as safe and effective in different applications [27]. Some have suggested that older adults are less receptive to hypnosis. However, studies involved older adults with positive results for hypnosis in pain [28], sleep disorders [29], and lumbar puncture related distress [30]. The feasibility of hypnosis in older people has been demonstrated in hospitalized [31] and home care populations [32]. While few studies included older persons with cognitive impairment [33], hypnotizability does not seem to change with age. Hypnosis appears to be particularly interesting in reducing pain, anxiety and medications in this population with higher susceptibility to adverse effects and drugs interactions.

### Specific aim and hypothesis

Since there is no study, to our knowledge, assessing the effect of hypnosis on fear of falling in a geriatric population, we conducted a feasibility trial to address if a hypnosis intervention would be feasible and acceptable, and explore effectiveness for fear of falling reduction, to inform a potential future large-scale study.

The primary aims of this study were to determine the feasibility and acceptability of a hypnosis intervention in older fallers. The exploratory aims were to assess the impact of hypnosis plus usual care (i.e., rehabilitation program) on fear of falling, based on the Perform-FES, other fear of falling scales, and other clinical outcomes including functional status, medications use, in-hospital falls, and length of hospital stay, compared to usual care alone.

## Methods

### Setting and participants

This single-center, two-arm, feasibility randomized controlled trial was conducted in a 296 bed acute care and rehabilitation geriatric hospital of Geneva University Hospitals (Switzerland), from January 17, 2019 to July 25, 2019 (NCT04726774). The study included hospitalized patients aged 65 years and over, admitted to a rehabilitation program specialized in falls and fracture risk assessment and management (“CHutes Et OsteoPoroSe” program, CHEOPS) that has been described elsewhere [20]. Exclusion criteria included patients with behavioral disorders, or who did not speak French, or lacked decision-making capacity. The participants should have their capacity of discernment to subscribe to the study, which mean they had the ability to understand the objectives of the study, what it implied and what were the risks and benefits, to finally decide if they wanted to participate or not. Capacity of discernment was assessed by the physician in charge of the patient. Behavioral disorders included opposition, aggressivity, or delirium, which would prevent the practice of intervention (rehabilitation and/or hypnosis). All these behavioral disorders were evaluated at inclusion by the hypnotherapist (i.e., subjective to the hypnotherapist). The target sample size was 30 participants (15 participants in each group). The study was approved by the Geneva Research Ethics Committee (2018–01550). All patients provided written informed consent before any study-related procedure.

### Randomization and blinding procedure

After consent, patients were randomized to either the intervention group or the control group. The randomization sequence was computer-generated (ratio 1:1) and concealed until patient enrolment. The nature of the intervention prevented us from blinding patients and the hypnotherapist to allocation. All clinical assessors, including physiotherapists and occupational therapists, were blinded to group allocation. All statistical analyses were performed by a blinded statistician (FRH).

### Interventions

The intervention consisted of two hypnosis session of about 30 min, provided weekly by a physician certified and trained in medical hypnosis (MPZB). MPZB is a medical doctor practitioner with university-level training in medical hypnosis and holds a diploma delivered by the Swiss Medical Society of Hypnosis. The intervention was provided in addition to the usual rehabilitation program. The participant was informed about medical hypnosis in term of general concepts and objectives of the study before inclusion. Hypnosis session were realized in the patients' room or in the physiotherapy room. Patient and hypnotherapist were face to face. If possible, all the hypnosis procedure was performed during walking, depending on the agreement and the physical status of the participant. During the consent interview, induction channels were explored, to prepare the first hypnosis session. The hypnosis procedure was divided into 4 phases: induction, trans hypnotic with walk perception alteration, post-hypnotic suggestion, and exit of hypnosis. Hypnosis was used as a communication tool adapted to the patient’s situation. During the trans hypnotic period, metaphors based on the patient’s personal history were used. Thus, the patient can experience his symptoms at another level of consciousness, non-analytical, non-rational, but in relation to sensoriality.

Both the intervention and control groups received the usual rehabilitation program, a multifactorial fall-and-fracture risk-based assessment and management intervention*,* which has been shown to be effective in improving physical parameters related to the risk of falls among high-risk oldest old patients [20]. This program includes an individually tailored intervention targeting each patient’s individual risk factors and impairments, including intensive physiotherapy for at least 2 weeks (i.e., focused on gait, balance and muscular function, in group and individual format and patient education on falls prevention). In this program, there is 5 weekly group sessions of 60-min duration and 3 to 5 weekly individual sessions of 30–45-min duration.

### Fear of falling assessment

The falls efficacy scale international (FES-I) was used to assess participant’s perceived fall risk by asking about concern about falling across a wide range of activities of daily living and social activities [23, 34]. The FES-I has excellent reliability (Cronbach’s α = 0.97, test–retest = 0.94) [23], and good psychometrics properties [35]. It comprises 16 items (housecleaning; dressing or undressing; preparing simple meals; bathing or showering; shopping; sitting or rising from a chair; walking up or down stairs; walking in the neighborhood; taking something above the level the head or from the ground; picking up the phone; walking on a slippery surface; visiting a friend or relative; walking in crowded places; walking on an uneven surface (with stones or holes); up or down a slope; and attending a social event). The total score varied between 16 (not worried) and 64 points (very worried).

The Perform-FES, a new scale derived from FES-I short version (7 items), but based on performance in real situations, was also used [25]. This scale was elaborated and validated for the purpose of the study, to better assess the fear of falling in older hospitalized patients who may have difficulties to report their concern about falling in specific daily-life tasks. Each situation (i.e., dressing or undressing: taking off one’s socks or dressing gown; bathing or showering: step into the shower or the bath, turn on the tap and get out; sitting and rising from a chair; walking up and down stairs: four steps; taking something from the ground; walking up and down a slope; and getting out: walk on a stony path and sit on a bench) was reproduced according to a standardized administration procedure, under the supervision of an occupational therapist. The total score varied between 7 and 28 points (1: not worried; 4: very worried). It had been shown previously that the Perform-FES had a good internal consistency (Cronbach's alpha coefficient = 0.78) and an excellent reliability (intraclass correlation coefficient = 0.94) in a hospitalized geriatric population [25]. This scale also revealed higher performance than other fear of falling scales in discriminating patients with severe functional limitations.

The other scales used to estimate the fear of falling were:

- The Activities-specific Balance Confidence scale [22, 36]. Patients are required to self-rate their degree of confidence in their balance associated with the performance of a series of daily living tasks. It was validated with high-functioning seniors. The simplified version includes 15 items, with a 4-category response format with descriptive anchors (i.e., 0: not at all confident, 1: slightly confident, 2: moderately confident, 3: very confident). Minimal score is 0, and maximal score is 45.
- The Geriatric fear of falling measure [21]. It contains 15 items to assess older adult’s fear of falling through psychosomatics symptoms, adopting a risk prevention and modifying behaviour, and not only with restriction activities [37]. Scores vary from 15 points (never concerned) to 75 points (always concerned).

Each scale was completed by each participant three times (week 0 (at baseline): before intervention; week 1 (during intervention): between the two hypnosis sessions; week 2 (at the end of the study): after all interventions). To avoid recall bias, scales were completed just after the standardized scenario of the Perform-FES, which mimics situations at risk of falling. If patients had difficulties in completing the questionnaire, the occupational therapist helped him.

### Other assessments

Demographics, comorbidities, cognitive, and nutritional assessment were assessed at baseline. Socio-demographic data included age, sex, weight(kg) and body mass index(kg/m^2^), place of living (home, residential home, or nursing home). The Cumulative Illness Rating Scale-Geriatric (CIRS-G) [38] estimated the number and the severity of comorbidities. Cognitive status was assessed by the Mini Mental State Examination (MMSE) [39], and the clock-drawing test [40]. Pain was assessed by the Visual Analog Scale (VAS), and depression by the mini Geriatric Depression Scale (mini-GDS) [41, 42]. The nutritional status was measured by the Nutritional Risk Score (NRS-2002) [43]. Number of drugs (total and specific medications such as anxyolitic and/or hypnotic drugs), physical performances (Short Physical Performance Battery (SPPB) [44]) and functional independence (Functional Independence Measure (FIM) [45]), were evaluated at baseline and at the final visit post intervention. SPPB is a composite physical performance assessment tool including a balance test, a gait speed test and a chair rise test. Poor performances on SPPB have been associated with in-hospital falls, injurious falls, and fractures in our population [46]. The FIM includes 18 items designed to assess the degree of assistance required for a person with a disability to perform basic life activities safely and effectively. It is widely used to determine the progress that patients make through programs of medical rehabilitation. The number of falls within 6 months preceding admission was collected by a nurse at admission based on self-report. In hospital falls were prospectively recorded until discharge using computer‐based incident report forms mandatorily completed after each fall by nurses and electronic patients’case notes or medical reports (i.e., through the integrated hospital information system).

### Analysis

Given the design of this study, we based the sample size on the feasibility objective, rather than on a formal power calculation to detect between-group difference for patient reported outcomes. A sample of 30 patients (15 per arm) was deemed large enough to provide useful information about feasibility. The sample was based on the same eligibility criteria that would be used in a future full-scale randomized controlled trial. Descriptive statistics were reported as mean ± standard deviation or median [interquartile range (IQR)] for continuous variables or number (percent) for categorical variables. The hypnosis and the control groups were compared at baseline using *t* tests, Wilcoxon rank sum tests or Fisher’s exact test as appropriate. All variables were tested for normality and appropriate transformations were used to transform non-normally distributed variables.

### Primary outcomes: feasibility and acceptability of intervention

Feasibility of recruitment and of the hypnosis intervention was assessed by measuring recruitment rate, retention rate, hypnosis adherence, and acceptance. Recruitment rate was analyzed by dividing the number of included participants by the number of weeks it took to include them. Retention rate was defined as the percentage of patients enrolled at baseline, who completed all follow-up measurements. Hypnosis adherence was measured by the proportion of the total number of sessions attended to the total number of sessions for which participants were enrolled to attend and by the proportions of participants who received all the sessions (i.e., 2 sessions). Adverse events (including abreactions, falls during hypnosis, headache and sleep disorders) were collected.

### Exploratory outcomes

Longitudinal data were analyzed according to the intention-to-treat (ITT) principle. Fear of falling, FIM and SPPB scores were analyzed using linear mixed-effects regression models, fitted using Stata “xtmixed” procedure, taking into account random effects (participant) and with the interaction group and visit entered in the models. These models took the repeated measure design of the study into account and allowed for a different number of observations within subjects. The same analysis was conducted in the per-protocol (PP) population (defined as participants who completed the intervention period and assessment visit) and in the subgroup of patients with a high concern of fear of falling (i.e., FES-I score ≥ 28/64, Perform-FES score ≥ 9/28) [47]. The incidence of inhospitals falls during hospital stay was analyzed using a negative binomial regression model. The length of stay in hospital (in days), the number of total medications at the end of rehabilitation program, and the presence or absence of anxiolitic and analgesic drugs, were compared between groups using *t* tests. A two-sided *p* value less than or equal to 0.05 was considered to indicate statistical significance. Data were analyzed using Stata version 16.1 (Stata Corp., College Station, TX) statistical software.

## Results

### Recruitment and participant flow

**Fig. 1:**
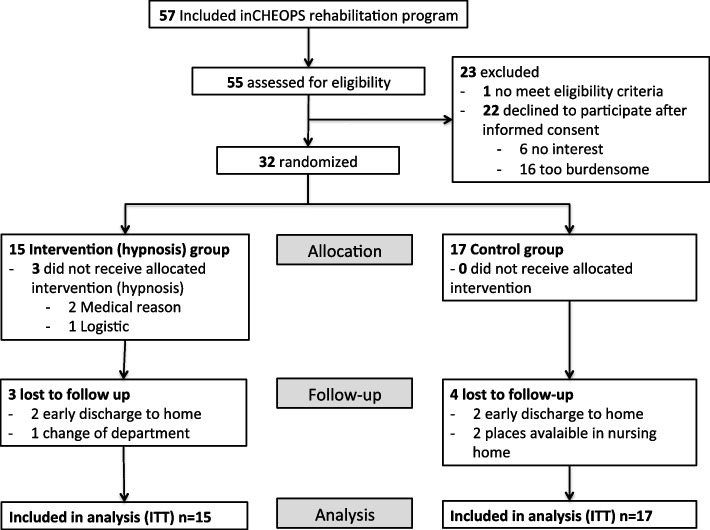
Flowchart of the feasibility randomized controlled trial

Among 57 patients admitted in the CHEOPS unit between January 17, 2019, and July 04, 2019 (168 days), 55 (96.5%) were assessed for eligibility. Of these, one did not meet inclusion criteria (delirium), and 22 (40%) declined to participate. The reasons for refusing to participate to the study were due to the nature of intervention in 6 cases and for other reasons in 16 cases (engagement, questionnaires, no interest). In total, 32 (58.2%) patients met eligibility criteria and provided informed consent. The recruitment rate was 1.3 patients per week (32 patients/24 weeks). After randomization 15 patients were included in the hypnotherapy group, and 17 in the control group. Seven patients were lost to follow-up (3 in hypnotherapy group, 4 in control group). The reasons were an early discharge from hospital in all cases (change of department (*n* = 1), transfer to a nursing home (*n* = 2) and return home (*n* = 4)). Enrolment and follow-up of patients are illustrated in Fig. 1.

### Baseline characteristics

Baseline characteristics of the study population are shown in Table 1. The mean age of the patients was 85.6 (± 5.8 SD) years, and 53% were women. Most of them (93.2%) lived at home before admission. The median score of MMSE was 25 [19;26] and 14 (43.8%) patients had a MMSE score ≤ 24. In terms of falls, only 3 (9%) patients reported no falls, while 18 (56%) reported 2 or more falls in the previous 6 months. Among the fallers, 22.7% had a fall related injury and 36.4% spent more than 30 min on the floor after a fall. At admission, the mean MIF score was 86/126 (± 19.1 SD) and the mean SPPB score was 5/12 (± 2.0 SD). Almost half of the included patients were taking analgesics (52.3%) and/or anxiolitic-hypnotic (43.2%) drugs at admission. The mean number of medications at admission was 8.8 (± 3.0 SD). The mini-GDS and NRS-2002 score were significantly different between both groups at admission (*p* = 0.003 and *p* = 0.025 respectively).

**Table 1:** Characteristics of patients at inclusion

Characteristic	Hypnosis group (*n* = 15)	Control group (*n* = 17)
Age, years	84.9 ± 5.9	86.1 ± 5.8
Sex, women	8 (53.3%)	9 (52.9%)
Place of living before admission		
Home	13 (86.7%)	16 (94.1%)
Residential home	2 (13.3%)	0 (0.0%)
Nursing home	0 (0.0%)	1 (5.9%)
Weight (kg)	67.1 ± 17.6	76.1 ± 16.4
Body mass index (kg/m^2^)	24.4 ± 6.3	28.0 ± 6.5
Cumulative Illness Rating Scale-Geriatric *[score range 0–56]*	15.4 ± 5.4	15.2 ± 5.0
Mini Mental State Examination *[score range 0–30]*	25 [21;26]	23 [18;27]
Score ≤ 24	5 (33.3%)	9 (52.9%)
Clock-drawing test *[score range 0–10]*	9 [7;10]	8 [7;10]
Mini-Geriatric Depression Scale [score range 0–4]*	2 [1;2]	0 [0;1]
Nutritional Risk Score 2002 [score range 0–7]*	3.3 ± 1.4	2.4 ± 0.9
Score ≥ 3	8 (53.3%)	4 (23.5%)
Visual Analog Scale *[score range 0–10]*	0 [0;2]	0 [0;0]
Number of drugs	8.5 ± 3.4	9.1 ± 2.8
Use of analgesic drugs	5 (33.3%)	7 (41.2%)
Use of anxiolitic drugs	6 (40.0%)	6 (35.3%)
Functional Independance Measure *[score range 18–126]*	89 ± 20.4	83 ± 18.1
Score < 110	10 (66.7%)	14 (82.3%)
Short Physical Performance Battery *[score range 0–12]*	5 ± 2.1	5 ± 1.9
Score ≤ 6	13 (86.7%)	12 (70.6%)
Number of previous falls (past 6 months)	2 [1;3]	2 [1;3]
≥ 1 fall with serious injury	5 (33.3%)	2 (11.8%)
≥ 1 fall with time on floor > 30 min	5 (33.3%)	6 (35.3%)
Length of hospital stay (days)	30 [26;36]	29 [24;41]
Length of rehabilitation program (days)	21 [15;21]	20 [14;21]

Data presented as mean ± standard deviation, median [interquartile range] or number (percent)

^*^There was a significant difference in the Mini-Geriatric Depression Scale (*p* = 0.003) and in the Nutritional Risk Score (*p* = 0.025) between the hypnosis group and the control group

At baseline, the mean scores of fear of falling scales were 32.3 ± 12.5 for the FES-I, 9.9 ± 3.1 for the Perform-FES, 44.7 ± 12.5 for the GFFM, 23.4 ± 8.4 for the ABC-S, without significant differences between the intervention and the control groups (*p* > 0.05 for all). Half (50%) of patients had a FES-I score ≥ 28/64, while 59% had a Perform-FES score ≥ 9/28, which are known thresholds above which fear of falling is a concern [47]. The two groups were comparable regarding physiotherapy adherence (9.5 ± 3.67 sessions in the intervention group vs 9.5 ± 4.14 sessions in the control group; *p* = 0.984).

### Primary outcome: feasibility and acceptability of hypnosis

Recruitment of 32 patients (our recruitment target), took 5.5 months. Retention rates were similar between the two groups: 10 patients (66.7%) in the hypnosis group successfully completed the follow-up (i.e., 15 days rehabilitation program and completed all scores and questionnaires) compared to 11 (64.7%) in the control group. The length of hospital stay and the length of the rehabilitation program did not differ between the two groups (*p* = 0.89 and *p* = 0.31 respectively) (Table 1).

In total, 23 sessions of hypnosis were delivered, with an adherence rate of 76.7% (95.8% after exclusion of 3 patients who did not receive any session). Four out of 15 patients in the hypnosis group did not have the full intervention (i.e., two hypnosis sessions), with no session in 3 cases, and only one session in 1 case. The reasons were medical (*n* = 2), logistic (*n* = 1), and refusal of the second session (*n* = 1). The mean duration time of the first and second sessions were 46.0 ± 13.0 min and 35.0 ± 8.2 min, respectively. The mean number of hypnosis sessions per patient was 1.5 ± 0.8. Fourteen out of 23 sessions of hypnosis were not performed while walking. The principal cause of non-walking hypnosis was patient's choice. The most used induction channels were by memory and kinesthesic. No adverse event related to the intervention was reported.

### Exploratory outcomes

Scores for the two groups at the different assessment visits are summarized in Table 2. In the ITT analysis, no significant randomization group × time interaction effects were found at both intermediate and end visits for any fear of falling outcomes (*p* > 0.05 for all). Also, no significant randomization group × time interaction effects were found at both intermediate and end visits for SPPB and FIM outcomes (*p* > 0.05 for all). Similar to the ITT analysis, neither the PP analysis nor the analysis in the subgroup of patients with a high fear of falling found any significant randomization group × time interaction effects for any fear of falling and functional outcomes. No significant effect was observed for other secondary outcomes, such as the number of drugs (*p* = 0.91), the presence or absence of anxiolitic (*p* = 0.18) and analgesic drugs (*p* = 0.70) at the end of hospitalization, or inhospitals falls experienced during hospital stay (IRR = 0.875; 95%CI 0.46–1.68; *p* = 0.689).

**Table 2:** Exploratory outcomes: scores at baseline, intermediate, and at the end of the study, for both groups

Scores	Group	Baseline visit (week 0)	Intermediate visit (week 1)	End visit (week 2)	P* (interaction group × time)
FES-I *[score range 16–64]*^a^	Hypnosis	33 ± 11	32 ± 11	27 ± 8	0.631
	Control	31 ± 13	31 ± 14	26 ± 10	
GFFM *[score range 15–75]*^a^	Hypnosis	45 ± 11	45 ± 9	45 ± 13	0.401
	Control	44 ± 14	44 ± 9	44 ± 11	
ABC-S *[score range 0–45]*^b^	Hypnosis	23 ± 8	26 ± 10	28 ± 10	0.611
	Control	24 ± 9	25 ± 9	28 ± 9	
Perform-FES *[score range 7–28]*^a^	Hypnosis	9.8 ± 2.7	9.7 ± 2.1	8.7 ± 2.7	0.117
	Control	10.1 ± 3.4	9.0 ± 2.8	8.1 ± 1.4	
SPPB *[score range 0–12]*	Hypnosis	4.8 ± 2.1	6.1 ± 2.9	6.4 ± 3.2	0.408
	Control	4.8 ± 1.9	5.2 ± 1.7	5.6 ± 2.4	
FIM *[score range 18–126]*	Hypnosis	88.9 ± 20.4	92.5 ± 17.0	97.1 ± 18.0	0.127
	Control	82.9 ± 18.1	94.5 ± 19.1	98.0 ± 20.4	

*FES-I* Falls Efficacy Scale-International, *GFFM* Geriatric Fear of Falling Measure, *ABC-S* Activities-Specific Balance Confidence Scale-Simplified, *Perform-FES* Performance-Fall Efficacy Scale, *SPPB* Short Physical Performance Battery, *FIM* Functional Independence Measure

^*^Interaction effect between group and time at end visit

^a^A higher score indicates a greater concern about falling

^b^A higher score indicates a lower concern about falling

In the study population as a whole, the SPPB score improved with the rehabilitation program (time effect, 1.2; 95% CI 0.6–1.8; *P* < 0.001), as did the FIM score (time effect, 11.5; 95% CI 7.1–16.0, *p* < 0.001).

## Discussion

Our results support the feasibility and acceptability of medical hypnosis in an inpatient geriatric rehabilitation population. We demonstrated a recruitment rate of 1.3 patients per week with a period of inclusion of 5.5 months for 32 patients and a good hypnosis adherence rate (95.8%). No adverse event of hypnosis was reported. Only one person refused the second session, without any adverse event reported. Exploratory outcomes were not able to support the effectiveness of hypnosis on fear of falling, self-efficacy and on functional performance. A larger randomized control study may be needed to assess the impact of hypnosis on fear of falling.

Concerning recruitment, there was no reluctance for hypnosis in our inpatient geriatric population. Only 11% (6/55) refused to participate in the study due to hypnosis intervention. One identified barrier to participation was multiple and self-administered questionnaires. We used several scales because, as discussed before, they not all similarly measure the fear of falling. The recruitment was acceptable (56%) with a rate of recruitment of 1.3 patients per week for 57 patients admitted in the rehabilitation program during this period. Adherence to the hypnosis intervention was relatively good, which suggests that the intervention was suitable for this particular sample of older patients. Retention rate was about 65%, most drop out cases were due to a request for an early return home (only 4 persons did not complete the entire rehabilitation program due to medical reasons), without a difference in the number of patients lost to follow-up between the two groups. These requests were related with a functional improvement allowing a return home and an early success of the rehabilitation program.

The study population was characterised by low physical performance (mean SPPB below 6) with high risk of falling and high fear of falling (mean ABC score < 50 [48] and mean FES-I > 28 [47]). Almost half of them (46.8%) had a Perform-FES score > 9/28. It has been previously shown that there was a correlation between SPPB and Perform-FES with a good ability for the Perform-FES to discriminate patients with several functional limitations. Moreover, functional limitations, as assessed by the SPPB, has been shown as an independent predictor of inhospital falls and fractures in our setting [49].

In studies evaluating hypnosis in rehabilitation, authors conclude that hypnosis could facilitate psychological and physical changes by reducing pain, fear or anxiety and increase patient’s motivation in rehabilitation [50–55]. To our knowledge, none of them concerned hypnosis and fear of falling or a specific geriatric population. Modalities of hypnosis were adapted to this inpatient geriatric population and could be useful for further studies, as short sessions lasting less than one hour, and induction channels adapted to the sensory impairments of patients. No adverse event and especially no falls were reported during hypnosis sessions. However, most of them (61%) differed from the initial planned procedure because they were not performed during walking. It was the patient’s choice in most cases and it could be explained, according to us, on the one hand by fear of falling and on the other hand by the misconception of loss of consciousness during hypnosis. The objective of hypnosis during walking was to strengthen the anchors when walking. To be useful, the association of positive emotions, good feelings and good sensations with walking needs to be repeated. Extending the use of hypnotic communication to the entire healthcare team would permit a continuation of the effect of hypnosis sessions.

The number of hypnosis sessions could be questioned. We chose two hypnosis sessions per patient for feasibility with only one hypnotherapist, and to be realistic in a two weeks rehabilitation program. It was possible that the hypnotic effect has a limited time effect [31, 56, 57]. In the review of Jensen and Pattersen about hypnotic treatment for chronic pain, almost all studies included a minimum of four sessions of hypnosis, and most of them encourage the additional practice of self-hypnosis [56]. Self-hypnosis could allow a longer effect of hypnosis. As already reported in a study focusing on hypnosis in older persons, hypnosis sessions could be shorter due to a decrease in attentional capacities, especially in case of cognitive impairment [32].

Although our study population reflects a geriatric population with low physical performance scores, high risk of falls and high fear of falling, we were not able to show the effect of hypnosis on fear of falling in this feasibility study. We did however demonstrate feasibility supporting the performance of a future more largely powered study. Scales of fear of falling had good validity and reliability, but their sensitivity to change is less studied. There is no longitudinal study yet concerning the new scale Perform-FES. Moreover, we could not show a reduction in medication use with hypnosis, which would have been an interesting benefit in this population with a high risk of drug-induced iatrogenic disease.

In the whole study population, patients improved their SPPB and FIM scores with the rehabilitation program. These results confirm the beneficial effects of a multifactorial fall-and-fracture risk assessment and management program, applied in a dedicated geriatric hospital unit, to improve fall-related physical performances and the level of independence in activities of daily living in high-risk patients [20].

### Strengths and limitations

The strengths of this study are the randomized controlled design and the standardized rehabilitation program, which may facilitate the design for a future larger randomized control trial. The feasibility and safety of hypnosis in this inpatient geriatric population with few refusals could encourage other studies with hypnosis in this specific old and frail population. The current study has limitations that need to be addressed. Some of these limitations are the small sample size and the number of lost to follow-up. However, the good recruitment rate, suggests the feasibility of a larger study. The number of patients lost to follow-up should be taken into account for further studies. In our experience, 20% (3/15) of the patients in hypnosis group did not receive hypnosis sessions at all. This could mask the potential effect of hypnosis on fear of falling. Other limitations are the number of secondary outcomes and the impossibility to conclude about the effectiveness of hypnosis on fear of falling due to a lack of power. The primary aim of this feasibility study was to evaluate feasibility rather than effectiveness. Regarding fear of falling, the number of outcome measures was extensive, especially given the absence of a gold standard measure in the hospitalized population. The FES-I scale is widely used in community-dwelling populations, since it has been validated in frail community-dwelling older adult with and without cognitive impairment [58] and reconciles the best sensitivity to change and the least missing data [58, 59]. Further works are still required to identify the best outcome measure to be used in our hospitalized population. Regarding patient’s characteristics, although it was a randomized study, there was difference at baseline for MMSE and NRS-2002 scores between the two groups. Both of these characteristics could influence falls and risk of falling but there was no difference in in-hospital falls and functional score between both groups during all the study. It worth to say that although cognitive disorders were not an exclusion criterion in our study, the mean MMSE score at baseline was relatively high (> 20/30). It could be explained by the inclusion criteria of the rehabilitation program and the need to consent to the study. For further larger studies, the prevalence of cognitive impairment should be considered because it can impact both adherence to the protocol and the response to the self-questionnaires. The Perform-FES scale seems to be a good alternative in this population as this scale has been developed in a hospitalized older population with approximately 40% of patients with cognitive disorders. The sensitivity to change of this scale is under evaluation by our group. Finally, as walking during hypnosis was almost not realized, we cannot conclude about this practice.

## Conclusion

In conclusion, this study confirmed that hypnosis is feasible and well accepted in an inpatient geriatric rehabilitation population. Based on these results, further pilot work should be performed with an increased number of hypnosis sessions and the use of hypnotic communication by the healthcare team in order to reinforce the hypnotic effect, before conducting a full-scale trial to conclude whether or not hypnosis is effective to reduce fear of falling. To identify the best outcome measures to be used in this full-scale trial to address fear of falling is also of utmost importance.

## Data Availability

The datasets used and analyzed during the current study are available from the corresponding author on reasonable request.

## Abbreviations

ABC: Activities-specific Balance Confidence scale
CHEOPS program: “ Chutes et OsteoPoroSe”
FES-I: Fall Efficacy Scale-International
FIM: Functional Independence Measure
GDS: Geriatric Depression Scale
GFFM: Geriatric Fear of Falling Measure
ITT: Intention-to-treat
MMSE: Mini Mental State Examination
NRS-2002: Nutritional Risk Score
PP: Per protocol
SPPB: Short Physical Performance Battery
VAS: Visual Analogue Scale
